# Does Public Health Influence Economic Performance? Investigating the Role of Governance and Greener Energies for the Case of China

**DOI:** 10.3389/fpubh.2022.864736

**Published:** 2022-03-29

**Authors:** Shaojie Huang, Tiansong Zhou, Chengying Xu, Jiahui Zheng

**Affiliations:** ^1^School of Humanities and Social Sciences, Guangxi Medical University, Nanning, China; ^2^School of Public Health, Guangxi Medical University, Nanning, China

**Keywords:** economic performance, public health expenditure, institutional quality, renewable energy, China

## Abstract

In the last few decades, the world has faced some natural issues, due to which economic growth faces a severe threat. Natural disasters like pandemic outbreaks and man-made disasters like pollution emissions are very frequent in the current times, which also influenced the economic growth, where the institutes could play a primary role in economic growth stimulation. This study aims to analyze the association of public health expenditures, institutional quality, renewable energy, and economic performance in China. This study uses quarterly data covering the period from 1996Q_1_ to 2020Q_4_ and employs various time-series estimating approaches. The Augmented Dickey-Fuller estimates asserted that all the variables are stationary at first difference. Also, the Bayer-Hanck combined cointegration validates that all the variables are cointegrated. Employing the three long-run estimators, i.e., Fully Modified Ordinary Least Square, Dynamic Ordinary Least Square, and canonical cointegrating regression, the results asserted public health expenditures and institutional quality (including government efficiency and political stability) significantly enhances economic performance in China. Whereas two indicators of corruption control and regulatory quality do not play any significant role in promoting the economic performance of China. On the contrary, renewable energy is found negatively associated with economic performance. Also, the Pair-wise Granger causality validates mixed causal associations between the study variables. As a developing and fossil energy-dependent economy, this study provides relevant policy implications for maintaining economic growth and rebalancing economic performance in China.

## Highlights

- The nexus of public health, renewables, and economic performance is analyzed.
- Quarterly data for China is utilized from 1996Q_1_ to 2020Q_4_.
- All the variables are found in the long-run equilibrium relationship.
- Health expenditures and institutional quality enhance economic performance.
- Renewable energy use adversely affects economic performance in the country.

## Introduction

The COVID-19 Pandemic outburst has raised many concerns about the influence of public health and epidemics on real economies. Public health is largely the discipline and art of how society aims to enhance health. Having good health is a number one asset of a strong nation, and it is a productive investment than consumption expenditure (1). In a report, Boyce and Brown (2) elaborated that the health sector is required for the stable and smooth functioning of the economy. Health systems in a country have a constructive impact on the economy's performance. It plays a necessary role in driving comprehensive and sustainable development. The well being of citizens contributes to social and economic prosperity that is important in the economy's long-term productivity. According to Wilkie and Young (3), a healthy population is a crucial driver of labor productivity, capital investment, and consistent economic growth. Besides, the definitive goal of economic policy is to enhance the health and well being of its people. Gross domestic product per capita or GDP per capita, a measure of well being, increases when the physical accumulation, technological improvements, and proportion of labor efficiency increases. The proportional correlation between income and health depicts that healthy populations have higher productivity and laborers are robust physically and mentally. They will have more incentives to invest in their education and skill development that benefits the economy wholly. They tend to save more, leading to an increase in the investment ratio. A healthy and educated labor force attracts foreign direct investment (4). Additionally, health isn't just related to the well being of nationals but also an investment that enhances the chances of future productivity. A healthy labor force works more proficiently, positively impacting economic performance (1). The economy's growth eliminates many issues, and good public health plays a considerable role in this economic growth.

Public health has been recognized as an important determinant of development with an economic return. At the same time, green growth is a necessary strategy for the sustainable development of a country. According to International Renewable Energy Agency (5), renewable energy or green energy is advantageous in improving the well being of humans and the country's Gross domestic product. The analysts suggest that increasing usage of renewable energy will reduce the fossil fuel demand in the future and increase capital-intensive renewables resulting in a positive impact on the gross domestic product (globally). Increasing industrialization has created many concerns environmentally and economically. Due to this reason, countries' health expenditure has risen, which has a significant influence on economic growth. Khan (6) determined that increasing renewable green energies reduces carbon and greenhouse gas emissions, leaving an encouraging image. Moreover, researchers of Harvard University claim that green energies and technology helps in reducing the human impact on climate change. Efficient energy and renewable energy consumption can improve the climate change concerns and public health (7). In the case of China, the government has made progress where people's standard of living and nutrition status has enhanced. The three geographical regions have different economic growth levels. And economic growth has a significant impact on the public's health (8). Further, China has grown its GDP forty times than in the 90s. It is expected that China will strengthen its ambitions and targets toward green energies and technology as it was featured in the past months (9). However, the Chinese public health expenditure shows fluctuations and is lower than the developed economies. There is a dire need for an efficient public health care system for the economy's proper functioning that helps improve the GDP (10).

The authors emphasize two models in this research study. The first model explains that the domestic health expenses, government effectiveness, renewable energy consumption, and regulatory quality influence the Chinese economy's Gross domestic product or GDP. The second model enlightens the effect of domestic health expenses, corruption control, renewable energy consumption, and political stability on the economy's Gross domestic product (GDP). Studies like (11–13) elaborated on the impact of governance, renewable energy consumption, political stability, and government effectiveness on economic growth and performance of the economy. An efficient form of government enhances economic growth and improves market efficiency. Likewise, political stability significantly enhances economic growth, enabling them to make durable and reliable economic policies for its progress.

GDP impacts the health of the economy, yet the focus of this article is on the reverse that there is a substantial impact of health on the performance of GDP. However, this study aims to contribute to the literature in the following ways. First, its main objective is to analyze the domestic health expenses (in two different models) for GDP in the case of China. Health is a necessary factor for sustainability, growth, and economic development, and previously it was not extensively analyzed in the studies. Instead, these studies focused on the environment, energy, and economic growth sides. Healthy people are efficient and are more productive for economic development (1). Therefore, the present research covers the gap by examining the public health influence on economic performance. The explanatory factors like government efficiency, political stability, and corruption control represent the role of governance in China, while renewable energy consumption is used for green energies to examine the impact of public health on GDP. This is well thought-out input in the literature. Second, Institutional quality variables have never been identified in prior literature for examining the association between public health and economic performance. This research is the pioneer in investigating the role of governance by taking institutional variables in analyzing public health's influence on the economy of China. China has become an active global leader in renewable energy production, and soon it will lead to deployment and investment in renewable energy internationally (14). This novel research will help analyze the effect of renewable energy, governance, and public health expenses on economic growth at the same time.

Now comes the organization of the article. The second section is about a brief review of literature related to the research where the association of public health and economic performance is discussed; section Data and Methodology is about data and methodology; section Results and Discussion deals with the results; and lastly, section Conclusion and Policy Implications is about conclusions and policy implications.

## Literature Review

This segment is about reviewing the literature related to the research study.

### Relationship Between Public Health and Economic Performance

To elaborate on the relationship between health and economic performance, it is necessary to understand the meaning of health. Health is not just the absence of illness; it is the ability of the people to develop their potential because it impacts the growth of the economy in several ways. Corresponding to human capital, health affects labor productivity and production (15). Health unswervingly impacts the level of productivity. As Barro (16) stated, given the labor working hours, physical capital, and experience, an enhancement in healthcare might increase labor productivity. It will also lower the mortality rates and diseases that directly impact the growth and development of the economy. The healthier the labor, the more efficient they will perform, directly proportional to economic prosperity. According to Bloom and Canning (1), health is the investment that increases the future productivity of individuals and the economy. The mechanism by which health affects the workers' productivity leads toward the growth of the economy of any country. The health market is superior, and the provision of health facilities can only be made by government intervention. In the case of China from the year 2000 to 2017, the authors analyzed that economic growth has a significant impact on the health of the public (8). They used a panel threshold regression model that depicts that public health enhances after the exceeded threshold level. There is heterogeneity in different regions due to different regional economic development. Likewise, individuals in good health are efficient economically. The more one is healthier, the more there will be saving rates. In their article, Bhargava et al. (17) examined the determinants of economic growth. The model depicted that life expectancy as a health indicator has significant effects on low-income economies generally because of nutrition level, diseases, and other factors like health infrastructure that affect health. Moreover, in the 1990's the public health experts and policy analysts recognized the correlation between macroeconomic indicators and health in the world bank development report (18). They emphasized that government can help enhance the health of the people of the country and prevent diseases that are obligatory for economic development. Investing in health has not only welfare effects but also boosts economic performance. Healthier people have higher efficiency as it directly contributes to labor productivity. It is commonly said that the growth of the economy alleviates many issues. When countries become rich, they acquire the resources to clothe, feed, and health facilities for their people. Therefore, health influences the performance of the economy positively, which is necessary for prosperity and poverty alleviation (1). Similarly, Strittmatter and Sunde (19) recommended that public health care facilities have a significant impact on the dynamics of health. Additionally, their outcomes suggested that this has a positive effect on aggregate national income. In a research study, Wu et al. (20) examined the measurement of health impact on economic growth in Asian regions. They used the quantile-on-quantile estimation technique to measure the health quantiles' influence on quantiles of growth of the economies. The findings were consistent with the literature that healthcare's positive and negative effects repeatedly occur when growth rises in the countries.

### Governance and Green Energies Affect the Performance of the Economy

For the sustainable development of a country, green growth is an exceptional strategy. In their article, Hussain et al. (21) investigated the impact of green technology and the environment on the gross domestic product (GDP) of the countries from the year 2000 to 2020. Their findings suggest that green technology has a substantial effect on green growth. High GDP economies must manage their economic activities to upsurge green growth for environmental protection. According to International Renewable Energy Agency (5), renewable energy or green energy is beneficial in improving the well being of humans and citizens' welfare beyond the GDP (welfare is another best alternative to GDP). The scholars found that utilizing low-carbon energy projects lessens the carbon emissions and scaling up these projects can further decrease the cost competitiveness (IRENA). The investment in renewable energy technologies will significantly impact the environment. Abolhosseini et al. (22) investigated that green technologies are vital for reducing environmental emissions. Qin et al. (23), Qin et al. (24) green energies help in limiting carbon emissions and China is increasing renewable electricity usage for achieving 2030 climate goals. Whereas, the authors discovered that GDP growth increases carbon dioxide emissions. Financial sustainability or higher GDP depends on energy usage because the economy's growth increases the energy level. Green technologies have ecological advantages and economic and social benefits alongside. Miśkiewicz (25), in their empirical findings, confirmed that the new green technologies increase the efficiency of energy and enhance the productivity of the economy. They applied the bibliometric analysis and Granger causality tests for estimation. The findings suggest that the government should play an important role in investing in green technologies to better the environment and economy. Moreover, the debates on the environmental Kuznets Curve highlighted that pollution could be controlled by the role of government and renewable energy consumption. The hypothesis states that there is a dynamic association between the economy's growth and environmental quality. Environmental governance has become important in achieving the green deal goals for sustainability. Simionescu et al. (26), in their research of European countries from the year 2006 to 2019, depict that corruption control, a proxy of governance effectiveness, in the long run, plays a vital role in enhancing environmental sustainability. Shahzad et al. (27) suggested that the role of government and institutional quality helps in achieving environmental and developmental sustainability.

The Chinese government plays a priority role in renewable energy investment. According to the Centre for strategic and international studies (CSIS), China has ambitious goals in the future for renewable energy to lead the world in the deployment and investment in the renewable energy sector (14). The empirical study in the case of China from the year 2000 to 2017 illustrates that the economic growth in the country has a considerable influence on the health of the public. They studied different regions, and the results were distinct due to their heterogeneity. Beside the extended literature, the summary of the literature review is provided in Table 1.

**Table 1:** Literature summary table.

**References**	**Discoveries**
Bhargava et al. (17 )	Depicted that life expectancy as a health indicator has significant effects on low-income economies
Bloom and Canning (1)	Health affects the workers' productivity leads toward the growth
Lustig (15)	Health affects labor productivity and production
Wilkie and Young (3)	Gross domestic product has a substantial impact on health outcomes
Strittmatter and Sunde (19)	Public health care facilities have a significant impact on health dynamics and aggregate national income
Chiu (14)	Economic growth in the country has a considerable influence on the health of the public
Khan (6)	Increasing renewable green energies reduce carbon and greenhouse gas emissions
Miśkiewicz (25)	Pollution can be controlled by the role of government and renewable energy consumption
Sahlian et al. (12)	An efficient form of government enhances economic growth and improves market efficiency
Wu et al. (20)	Consistent with the literature that healthcare's positive and negative effects repeatedly occur due to growth variability in the countries
Shahzad et al. (27)	Government helps in attaining economic and environmental stability
Qin et al. (23, 24)	Green energies play a significant role in mitigating environmental pollution i.e., carbon and GHG emissions
Niu et al. (8)	Economic growth has a significant impact on the public's health
Hussain et al. (21)	Green technology and energy have a substantial effect on green growth. High GDP economies manage their economic activities to upsurge green growth for environmental protection

## Data and Methodology

### Data and Model Specifications

Following the prior literature and the study's objectives, this study uses seven variables. The focus variable is economic performance and captured by gross domestic product (GDP) since GDP measures the economy's health by considering production, consumption, investment, and expenditures, among others. Besides, the literature widely used GDP as an appropriate representative of economic performance (28, 29). Thus, this study also proxied economic performance *via* GDP following these studies. On the other hand, this study used public health—captured by public health expenditures (HE). The quality of health could be better assessed *via* expenditures on health, where better health quality could enhance economic performance. In order to stabilize economic growth and performance, most of the developing economies rely on fossil fuel energy consumption, which further fuels environmental degradation (30, 31). However, scholars claimed that using renewable energy could help rebalance economic growth and environmental quality (32, 33). Therefore, this study uses renewable energy consumption (REC) as an explanatory variable to analyze its impact on economic performance.

Moreover, the governance or institutional quality could play a substantial role in maintaining economic performance due to rule and policies implications. In this sense, the current study also aims to investigate the impact of institutional or governance quality, which could be represented *via* four indicators, i.e., government effectiveness (GEF), regulatory quality (RQ), corruption control (CRC), and political stability and violence control (PSV). Quarterly data for all the variables is obtained for China, covering the period from 1996Q_1_ to 2020Q_4_. Variables specifications and data sources are provided in Table 2.

**Table 2:** Variables specification and data source.

**Variable**	**Specification**	**Data source**
GDP	The monetary value of all complete services and goods, measured in constant US$ 2015.	https://databank.world-bank.org/source/world-development-indicators
HE	Domestic healthcare spending as a proportion of the economy (as measured by GDP, i.e., % of GDP).	http://apps.who.int/nh-a/database
REC	The percentage of renewable energy in total final energy use and measured as % of total final energy consumption.	https://databank.world-bank.org/source/environ-ment-social-and-governance-(esg)-data
GEF	Measures perceptions of public services quality, the civil service's independence from political constraints, the policy development and execution quality, and the reliability of the government's commitment to such policies, measured as a percentile.	www.govindicators.org
RQ	Measures public perceptions of the government's capacity to establish and enforce effective rules and regulations that allow and support the development of the private sector, measured as a percentile.	www.govindicators.org
CRC	Measures public perceptions of the amount to which public authority is used for personal benefit, encompassing both grand and petty corruption and “capture” of the state by elites and private interests, measured as a percentile.	www.govindicators.org
PSV	Perceptions of the possibility of political instability and/or politically motivated violence, including terrorism, are measured as a percentile.	www.govindicators.org

Following Niu et al. (8) and Hussain et al. (21), this study constructed the following two models:

Model 1

$$\begin{array}{l} GDP_{t}=f\left(HE_{t},REC_{t},GEF_{t},RQ_{t}\right) \end{array}$$

Model 2

$$\begin{array}{l} GDP_{t}=f\left(HE_{t},REC_{t},CRC_{t},PSV_{t}\right) \end{array}$$

The primary reason for taking the governance quality in two models is to tackle the issue of model bias. Model 1 demonstrates that GDP is the function of HE, REC, GEF and RQ, while Model 2 illustrates that GDP is the function of, HE, REC, CRC, and PSV. To empirically analyze the models, it should be transformed into regression form, expressed as follows:


(1) $$\begin{array}{l} GDP_{t}=\alpha_{1}\;+\;\alpha_{2}HE_{t}\;+\;\alpha_{3}REC_{t}\;+\;\alpha_{4}GEF_{t}\;+\;\alpha_{5}RQ_{t}\\ \;+\;\varepsilon_{t} \end{array}$$

(2) $$\begin{array}{l} GDP_{t}=\beta_{1}\;+\;\beta_{2}HE_{t}\;+\;\beta_{3}REC_{t}\;+\;\beta_{4}CRC_{t}\;+\;\beta_{5}PSV_{t}\\ \;+\;\varepsilon_{t} \end{array}$$

The above equations reveal that GDP is economic performance, HE is public health expenditures, REC designates renewable energy consumption, GEF stands for government efficiency, RQ indicates regulatory quality, CRC represents corruption control, and PSV stands for political stability in China. Besides, α′*s* and β′*s* are the slopes, where α_1_ and β_1_ are the intercepts of Equation (1) and (2), respectively. The subscript “*t*” indicates the time period, while ε is the component for random error of the model.

### Estimation Strategy

#### Descriptive Statistics

The first step to empirical investigation is to compute descriptive statistics. Descriptive statistics helps summarize the data by providing the mean, median, and range values, including the minimum and maximum of each variable. Additionally, the standard deviation is also evaluated, which indicates fluctuation of observation from the mean value. In addition, statistics for skewness and Kurtosis are also provided to demonstrate the normality distribution of each variable under study.

#### Stationarity Testing

Following the computation of descriptive statistics for data under examination, this research proceeds to test for stationarity or the existence of unit root in the time variables. We used the Augmented Dickey-Fuller (ADF) unit root test in this case. The ADF unit root test introduced by Dickey and Fuller (34) is efficient because it addresses the serial correlation challenge. Since this test manages more complicated models, it is regarded as more effective than the conventional Dickey-Fuller unit root test. In Equation (3), the generic form of the ADF unit root test might be given as follows:


(3) $$\begin{array}{r} y_{t}=\;\gamma \;+\;\phi t\;+\;\mu y_{t-1}\;+\;e_{t} \end{array}$$

For analysis of the above equation, the ordinary least square (OLS) approach could be used, expressed as follows:


(4) $$\begin{array}{r} \Delta y_{t}=\;\left(\mu -1\right)y_{t-1}\;+\;\gamma \;+\;\theta t\;+\;e_{t} \end{array}$$

Aside from the basic equation form, it is worth mentioning that the chosen ADF unit root test comes in two varieties: with the trend and with the intercept. Furthermore, the null proposition of the ADF unit root test is that the unit root exists in a time series.

#### Bayer-Hanck Combined Cointegration Test

After identifying the stationary or non-stationary behavior of data, this study investigated the cointegration correlation between different variables under consideration. In this sense, the current study used the Bayer-Hanck combined cointegration test, which combines the cointegration tests of Engle and Granger (35), Johansen (36) Banerjee et al. (37), and Boswijk (38). However, if such specifications are used independently, the cointegration analysis may produce inconclusive findings due to the explanatory power features of these tests (27). As a consequence, we used the combined cointegration test technique described by Bayer and Hanck (39) to improve the cointegration analysis power and overcome doubtful or ambiguous estimations. As per Shahzad et al. (27), this test includes all preceding cointegration tests in a combined method and offers conclusive and consistent results using Fisher F-statistics. Furthermore, this test necessitates a distinct sequence of integration, i.e., I(1). As a null hypothesis, the assumption implies no cointegration between the study variables. However, this null proposition might be rejected if the predicted values are significant at any significance level, i.e., 1, 5, or 10%. In general, the Bayer-Hanck cointegration Fisher's standard equation is as follows:


(5) $$\begin{array}{r} EG-J=\;-2\left[\ln\left(P_{EG}\right)\;+\;\ln\left(P_{J}\right)\right] \end{array}$$

(6) $$\begin{array}{l} EG\;-\;J\;-\;Ba\;-\;Bo\\ \;=\;-2[\ln(P_{EG})\;+\;\ln(P_{J})\;+\;\ln(P_{Ba})\;+\;\ln(P_{Bo})] \end{array}$$

Where Equation (6) designates that *P*_*EG*_, *P*_*j*_, *P*_*Ba*_ and *P*_*Bo*_ are probability estimates for Engle and Granger (35), Johansen (36), Banerjee et al. (37), and Boswijk (38) cointegration tests, accordingly.

#### Long-Run Estimations

The long-run connection between variables has been established using the Bayer and Hanck (39) combined cointegration techniques. This enables the present research study to evaluate the effects of each explanatory variable, namely HE, REC, GEF, RQ, CRC, and PSV, on China's economic performance. We must employ an appropriate and impartial estimator(s) in this sense. Hence, we applied three long-run estimation methodologies, as suggested by Khan et al. (40). Among these methods are Pedroni (41) dynamic ordinary least square (DOLS), fully modified ordinary least squares (FMOLS), and Park's (42) Canonical Cointegrating Regression (CCR). The two aforementioned techniques use distinct methodologies, namely non-parametric (FMOLS) and parametric (DOLS). Since these estimators have greater effectiveness in dealing with serial correlation and endogeneity concerns, they are robust measures of long-run estimates. Also, the DOLS operator effectively estimates time series since it addresses the non-stationarity issue. Both FMOLS and DOLS are expressed in the equation form shown below:


(7) $$\hat{\emptyset }=\left[\begin{array}{c} \alpha \\ \hat{\beta } \end{array}\right]=\;{\left(\sum_{t=2}^{T}Z_{t}{\overset{\overset{´}{’}}{Z}}_{t}\right)}^{-1}\left(\sum_{t=2}^{T}Z_{t}y_{t}^{\;+\;}-T\left[\begin{array}{c} {\hat{\theta }}_{\overset{\overset{´}{’}}{12}}^{\;+\;}\\ 0 \end{array}\right]\right)$$

Where Equation (7) is the standard form of FMOLS, employing $Z_{t}=\overset{´}{\left({\overset{´}{X}}_{t},{\overset{´}{D}}_{t}\right)}$. In studying the FMOLS approximation, the long-run covariance matrix is essential.


(8) $$\begin{array}{c} y_{t}=\;{X^{\prime }}_{t}\beta \;+\;{D^{\prime }}_{1t}\gamma_{1}\;+\;\overset{r}{\underset{j=-q}{\sum^{\text{​}}}}△{X^{\prime }}_{t\;+\;j}\sigma \;+\;v_{1t} \end{array}$$

Where Equation (8) reveals the DOLS standard form, including the expansion of cointegration regression while allowing for lags $△{\overset{´}{X}}_{t}$ and leads due to the orthogonal error term cointegration equation. The DOLS estimator implies that by combining the *r* leads and *q* lags of different regression coefficients, the long-run connection between *e*_1*t*_ and *e*_2*t*_ maybe detected.

As stated previously, the CCR estimating technique is entirely a regression-based approach. However, this strategy is economical and plays an important role in resolving the linear regression component (43). As a result, the determination of precise lags and lead orders is one of the most significant challenges for the under-discussion technique. In general, the CCR estimators might be expressed in the form of an equation below:


(9) $$y_{t}{}^{*}={\overset{´}{\beta }}_{pq}z_{pqt}^{*}\;+\;\mu_{pqt}^{*}$$

Where both $y_{t}^{*}$ and $z_{pqt}^{*}$. The above equation is the stationary conversion of *y*_*t*_ and *z*_*pqt*_, respectively.

#### Pairwise Granger Causality Test

The regression method is commonly used to depict “simple” correlation, whereas Granger (44) claimed that causality in economics could be assessed by measuring the means of predicting values of one time-series given prior values of another time-series. Nonetheless, the long-run estimators provide important empirical results, yet these approaches are limited in demonstrating the causal specifications between GDP and regressors. Therefore, the current study used the Granger causality test, which was established by Granger (44). This test is efficient since it can be run on either I(0) or I(1) data. To assess the null hypothesis of the stated test, which states that *z* does not Granger cause *x*, it is necessary to determine the right lagged values of *x* to incorporate in a univariate autoregressive model of *x*.


(10) $$\begin{array}{r} x_{t}=\theta_{1}\;+\;\theta_{2}x_{t-1}\;+\;\theta_{3}x_{t-2}\;+\;\ldots \;+\;\theta_{m}x_{t-m}\;+\;\varepsilon_{t} \end{array}$$

The autoregression is expanded by incorporating lagged values of *z*, given below:


(11) $$\begin{array}{c} \begin{array}{l} x_{t}=\theta_{1}\;+\;\theta_{2}x_{t-1}\;+\;\theta_{3}x_{t-2}\;+\;\ldots \;+\;b_{p}z_{t-p}\;+\;\ldots \\ \;+\;b_{q}z_{t-q}\;+\;\theta_{m}x_{t-m}\;+\;\varepsilon_{t} \end{array} \end{array}$$

The lagged values of z, which are singly significant, based on their *t*-statistics, are preserved in this approach, as they cumulatively offer the power of prediction to the regression based on the *F*-test. In this case, *p* is the shortest lag length, and *q* is the longest, such that the lagged value of *z* is important in the preceding regression augmentation. If no lagged *z* values are preserved in the regression, this will accept the null proposition that *z* does not Granger cause *x*.

It is noteworthy that all the equations used in this study are adapted from the original studies of the authors' proposed tests, as mentioned with each specification employed.

## Results and Discussion

In this section of the article, results and their elucidation are discussed. Table 3 represents the descriptive statistics of the variables used in the study. Table 4 shows the unit root test results to analyze whether the outcomes are stationary or not, Table 5 is about the cointegration results Bayer-Hanck (39), Tables 6, 7 are empirical long-run estimates of the Model 1 & 2, and Table 8 shows the pairwise granger causality results of the study. Then a summary of the discussions is briefed at the end.

**Table 3:** Descriptive statistics.

	**GDP**	**HE**	**REC**	**CRC**	**GEF**	**PSV**	**RQ**
Mean	7.26E + 12	1.942360	18.27155	42.55504	59.12864	31.99202	44.20462
Median	6.46E + 12	1.879487	14.00580	44.78902	58.03561	30.46017	44.27178
Maximum	1.49E + 13	3.018888	30.53718	53.40818	73.23947	44.14894	50.97087
Minimum	2.01E + 12	0.967205	11.33820	33.17073	43.16940	25.59242	34.18367
Std. Dev.	4.17E + 12	0.802624	7.232003	5.861273	7.281592	4.543164	4.396306
Skewness	0.399982	0.099237	0.737315	−0.125500	0.332895	0.490237	−0.492710
Kurtosis	1.791468	1.306116	1.809698	1.642881	2.382994	2.054428	2.549348
Sum	7.26E + 14	194.2360	1827.155	4255.504	5912.864	3199.202	4420.462
Observations	100	100	100	100	100	100	100

**Table 4:** Unit root testing.

**Variables**	**Augmented Dickey-Fuller**	
	**I(0)**	**I(1)**
GDP	−1.6006	−1.8869*
HE	1.2283	−2.2454**
REC	−1.5568	−4.6369***
CRC	0.1912	−1.9884**
GEF	1.7563	−2.3901**
PSV	−0.0092	−4.1098***
RQ	0.2917	−4.5308***

Significance is indicated by 10, 5, and 1% through ^*^, ^**^, and ^***^. I(0) is for level, and I(1) is for the first difference.

**Table 5:** Bayer-Hanck cointegration analysis.

**Engle-granger (EG)**	**Johansen (J)**	**Banerjee (Ba)**	**Boswijk (Bo)**
−1.8174	78.8175***	−0.7939	104.6554***
EG-J		EG-J-Ba-Bo	
55.3393***		110.6928***	

Significance is indicated by 10, 5, and 1% through ^*^, ^**^, and ^***^.

**Table 6:** Empirical results of model-1.

**Dep. Var. GDP**	**Coefficients [Std. error]**		
	**FMOLS**	**DOLS**	**CCR**
HE	0.339*** [0.0354]	0.314*** [0.0472]	0.339*** [0.0318]
REC	−0.029*** [0.0034]	−0.032*** [0.0043]	−0.029*** [0.0032]
GEF	0.027*** [0.0028]	0.027*** [0.0029]	0.027*** [0.0025]
RQ	0.0094 [0.0032]	0.0001 [0.0033]	0.0010 [0.0030]
*Constant*	27.693*** [0.2219]	27.797*** [0.2537]	27.684*** [0.2279]

Significance is indicated by 10, 5, and 1% through ^*^, ^**^, and ^***^. The standard error is provided in the brackets.

**Table 7:** Empirical results of model-2.

**Dep. Var. GDP**	**Coefficients [Std. error]**		
	**FMOLS**	**DOLS**	**CCR**
HE	0.521*** [0.0921]	0.513*** [0.0577]	0.522*** [0.0858]
REC	−0.037*** [0.0104]	−0.036*** [0.0065]	−0.037*** [0.0098]
CRC	−0.0025 [0.0064]	−0.0018 [0.0040]	−0.0022 [0.0059]
PSV	0.018*** [0.0054]	0.014*** [0.0033]	0.018*** [0.0049]
*Constant*	28.619*** [0.1497]	28.717*** [0.0938]	28.623*** [0.1479]

Significance is indicated by 10, 5, and 1% through ^*^, ^**^, and ^***^. The standard error is provided in the brackets.

**Table 8:** Pairwise granger causality tests.

**Null hypothesis**	**F-statistics**	**Prob**
HE ⇏ GDP	35.8618***	4.E-08
GDP ⇏ HE	15.0445***	0.0002
REC ⇏ GDP	195.256***	7.E-25
GDP ⇏ REC	29.1418***	5.E-07
GEF ⇏ GDP	22.6830***	7.E-06
GDP ⇏ GEF	1.26342	0.2638
RQ ⇏ GDP	26.2672***	2.E-06
GDP ⇏ RQ	3.38463*	0.0689
CRC ⇏ GDP	160.922***	3.E-22
GDP ⇏ CRC	7.88824***	0.0060
PSV ⇏ GDP	76.6741***	7.E-14
GDP ⇏ PSV	9.74871***	0.0024

Significance is indicated by 10, 5, and 1% through ^*^, ^**^, and ^***^.

### Descriptive Statistics and Unit Root Tests

As the first step of the analysis, the descriptive statistics of the research show the average values of Gross domestic product (GDP), Health expenditure (HE), renewable energy consumption (REC), Corruption control (CRC), Government efficiency (GEF), political stability (PSV), and regulatory quality (RQ). The median, maximum, and minimum values are also made known in the table. The median values are somehow nearby to their average values depicting the balancing point of the data. The standard deviation represents the volatility of the data and how much data spread around its mean values. Skewness and Kurtosis illustrate the normality and symmetry of the data, whether the data is normal or not. They range from + 2 to −2 and + 7 to −7 for skewness and Kurtosis, respectively (45). The skewness and values of Kurtosis of the research variables range between the above-mentioned range. The overall values of the data describe that the data is symmetrical and normally distributed.

After the descriptive analysis of the data, the stationarity tests results of the data in the time series are shown in Table 4 of this section below. Several financial and economic series show trending behavior in the mean. In added words, they have unit roots and are non-stationary in the mean values. Unit root tests are applied to determine the first difference stationarity in time series analysis. Usually, the non-stationary trendy data depicts that there is some association in the long run. Additionally, the unit root is also termed as the pre-testing test for cointegration analysis in the econometric time-series study. They called unit root because, in the null hypothesis, the root is equal to one (46). The Augmented Dickey-Fuller test is applied for testing unit root for the residuals. The test findings depict that the data is stationarity at first difference. Renewable energy consumption, political stability, and regulatory quality are stationary at a one percent level of significance, health expenditure, corruption control, government efficiency are significant at a five percent level of significance, whereas only GDP is significant at a ten percent level of significance. The coefficients of the first difference are negative but statistically significant. The more the negative value depicts a stronger unit root, the more the null hypothesis will be rejected.

### Bayer-Hanck's Cointegration

Bayer-Hanck's (39) cointegration analysis is used to investigate whether the research variables are cointegrated in the long run and infer causality. The conventional regression analysis does not infer causation. In Table 5, the Bayer-Hanck test is applied to determine the cointegration between the variables. Table 5 of the results discussion shows Bayer-Hanck cointegration tests outcomes along with EG-J and EG-J-Ba-Bo. For improving the influence of the cointegration test, the new test is based on combining the (35) (EG), (36) (J), (37) (Ba), and (38) (Bo) tests. The previous cointegration tests frequently reject one while the other does not reject, thereby complicating the overall interpretation. The traditional Bayer-Hanck cointegration was anew projected by Bayer-Hanck (39). The new test combines different cointegration tests for more decisive, reliable, and robust findings. The combined EG-J value shows that we will reject the null hypothesis at a 1 percent significance level. The value of value EG-J-Ba-Bo shows a significant value at 1 percent, rejecting the null hypothesis. The overall statistical findings of this new Bayer-Hanck (39) cointegration test show that there is cointegration among the variables in the long run. Thus, there is an influence of public health expenditure (other explanatory variables alongside) on the economy's performance. Therefore, we move toward the empirical result discussion in the succeeding tables.

### Long-Run Estimates

The objective of the research is to investigate the influence of health on the economy's performance. The previous results depicted that there is cointegration among the variables in the research study. The long-run estimates in the following tables for both models are based on three cointegration estimates, i.e., Fully modified ordinary least squares (FMOLS), dynamic ordinary least squares (DOLS), and canonical cointegrating regression (CCR). These estimators perform better than other econometric estimators. Tables 6, 7 represent the empirical outcomes of Model 1 and Model 2.

In model 1, the coefficient values of health expenditure, renewable energy consumption, and government efficiency are statistically significant at a one percent level of significance. The long-run estimates are positive and statistically significant except for renewable energy consumption, which is negative and statistically significant in the long run. The findings of the first model depict that health expenses and government efficiency positively influence the economy's performance, i.e., the GDP. A percentage increase in these variables will improve the performance of the economy. Likewise, renewable energy consumption has a negative but significant impact in the long run. The results are similar across the three estimators. In model 2, the coefficients of health expenditure and political stability positively influence the gross domestic product, i.e., economic performance. Renewable energy consumption has a negative but statistically significant connection to the country's economic performance. Correspondingly, corruption control has negatively related to the performance of the economy. The outcomes are identical across all three cointegration estimates.

The findings of both models (1 & 2) show that expenditure on public health has a positive influence on the economy's performance. Improved public health increases labor productivity, and the economy will perform better. Statistically significant impact on the dynamics of health has a positive effect on aggregate national income, i.e., the economic growth (19) & (20). Moreover, renewable energy confirms an inverse impact on the economy's performance as the results show there is no proportional direction between renewable energy consumption and economic growth, i.e., GDP. Several authors have explained the asymmetrical relation between green energy and the economy's economic growth (47) & (48).

### Pairwise Granger Causality Test

Causality is termed cause and effect between variables. The pairwise granger causality test determines whether one variable is predictable over the other variable in the model. In simple words, it is applied to examine causality between two variables (variable pair). Granger causality is not a real casual association among variables, but it is an estimation method that assists in forecasting the other variable better (49). For instance, if any two variables are cointegrated, then one Granger causes the other, or it can be *vice versa*.

The null hypothesis of the granger causality states that there is no Granger cause (⇏) between the variables. The hypothesis is rejected when the value is less than the significant level either it is at 1, 5, or 10% level of significance. The 12 pairs of variables are modeled in the research for granger analysis. Two variables (pair) are analyzed to determine their interaction. The f-statistics and probability values of the variable pair in Table 8 shows that the values are statistically significant, and the null hypothesis is rejected except when GDP ⇏ GEF. The test findings represent that is causality among the variables in predicting the behavior of the other variable. The findings suggest that most of the variables pair have bi-directional Granger causality. There is bi-directional causality between HE and GDP, GDP and HE, REC and GDP, GDP and REC, RQ and GDP, GDP and RQ, CRC and GDP, GDP and CRC, PSV and GDP, & GDP and PSV. For illustration, the health expenditure and gross domestic product have bi-directional causality. The response in GDP occurs due to changes in health expenses, and health expenses of the public vary due to changes in the GDP. Likewise, a change in political stability causes a change in the GDP of the economy and *vice versa*. All pairs in the model are Granger cause except the 6th model pair GDP and GEF. These variables have unidirectional causality that improvement in government efficiency causes a change in the GDP while there is no granger cause *vice versa* here. This means that changes in GDP have not helped improve the government efficiency in the country.

### Summary Discussion

The descriptive statistics of the data represent that the variables are balanced with normal distribution. At that point, determine the stationarity of the variables by Augmented Dickey-Fuller tests. The variables showed stationarity in the first difference. The negative values depict that there is stronger existence of unit root and lead to rejection of the null hypothesis. Later, for cointegration analysis, a combination of cointegration methods is applied for examining the association between the variables in the long run (39). This newly proposed method gave reliable estimates that led to the conclusion that there is cointegration in the long run. The empirical examination of both models showed that improved public health could increase labor productivity, and the economy will perform better. The long-run approximations were identical in all three (FMOLS, DOLS & CCR) cointegration estimates. Enhancing the public health expenses increases economic development. The research findings showed consistent results with (19, 20, 50). At the same time, there is a negative but statistical association between renewable energy and the economy's Gross domestic product. These findings were consistent with literature as presented by Baz et al. (48), Chen et al. (51), and (47). According to the researchers, due to the feedback hypothesis, energy consumption has an inverse impact on economic performance in developed economies, while due to the conservation hypothesis, it has a negative association in poorer economies. After the cointegration analysis, the last step of analysis is Granger causality. The pairwise Granger causality illustrates that public health expenditure and the role of governance can help improve the economy's performance. The overall results reveal that there is an existence of Granger cause among the variables that help predict the other variable.

## Conclusion and Policy Implications

To conclude all, public health is a topic of concern for past years. Health reforms in various cities of China have made the country powerful in economic development (50). This article argues whether there is the influence of public health on economic performance and the government plays part in achieving economic development. Theoretically, the role of governance and green technologies are partners in achieving economic development. The research, however, applies cointegration and granger causality to determine this relationship. We emphasized two models in this research study. The first model comprises domestic health expenses, government effectiveness, renewable energy consumption, and regulatory quality while the second model demonstrates the effect of domestic health expenses, corruption control, renewable energy consumption, and political stability on the GDP of the economy.

The findings of the research were consistent with (19, 47, 50, 51). First increasing the expenditure on public health will help in boosting the performance of the economy. The findings are backed by this statement. The better the labor the more competently they will perform which is directly proportional to economic prosperity. For the reason that certainly, the health impacts the performance of the economy which is essential for prosperity, and it is a priceless asset of every nation. Second, according to the results, renewable energy consumption and economic performance (GDP) have an inverse relationship. The negative relationship between them reveals that the role of renewable energy is lesser than other factors of development. According to researchers, the negative association occurs usually in those countries where there is no role of institutions. Third, the effective form of government and political stability impact the quality and improvement in a sustainable form of government (26).

The implications and suggestions include the public health development in the country and green technology with proper infrastructure sideways can be useful in achieving the long-term green goal objectives and sustainable progress. These cannot be attained without government intervention with effective laws, regulations, and policies that expressively enhance economic growth that enables to make durable and reliable economic and environmental strategies for the progress of the country. These results have vital significance in policymaking for promoting economic development efficiently. Further, this might provide insights for academics to apply this analysis to other countries, and different variables and economic indicators can be used to observe the relationship.

## Data Availability Statement

The datasets presented in this study can be found in online repositories. The names of the repository/repositories and accession number(s) can be found in the article/supplementary material.

## Author Contributions

SH: idea, data, literature, concept, introduction, and funds. TZ: editing, reviewing, writing methodology, and software. CX: conclusion, supervision, visualization, and estimates. JZ: results and discussion, introduction, and preparing draft. All authors contributed to the article and approved the submitted version.

## Conflict of Interest

The authors declare that the research was conducted in the absence of any commercial or financial relationships that could be construed as a potential conflict of interest.

## Publisher's Note

All claims expressed in this article are solely those of the authors and do not necessarily represent those of their affiliated organizations, or those of the publisher, the editors and the reviewers. Any product that may be evaluated in this article, or claim that may be made by its manufacturer, is not guaranteed or endorsed by the publisher.
